# Fluid Structure Interaction on Paravalvular Leakage of Transcatheter Aortic Valve Implantation Related to Aortic Stenosis: A Patient-Specific Case

**DOI:** 10.1155/2020/9163085

**Published:** 2020-05-04

**Authors:** Adi A. Basri, Mohammad Zuber, Ernnie I. Basri, Muhammad S. Zakaria, Ahmad F. A. Aziz, Masaaki Tamagawa, Kamarul A. Ahmad

**Affiliations:** ^1^Department of Aerospace Engineering, Universiti Putra Malaysia, Serdang 43400, Malaysia; ^2^Aerospace Malaysia Reaserch Center (AMRC), Universiti Putra Malaysia, 43400 Serdang, Selangor, Malaysia; ^3^Department of Aeronautical and Automobile Engineering, Manipal Institute of Technology, Manipal Academy of Higher Education, Manipal 576104, India; ^4^Fakulti Kejuruteraan Mekanikal, Universiti Teknikal Malaysia Melaka, Hang Tuah Jaya 76100, Durian Tunggal, Melaka, Malaysia; ^5^Department of Medicine, Universiti Putra Malaysia, Serdang 43400, Malaysia; ^6^Department of Biological Function and Engineering, Kyushu Institute of Technology, Kitakyushu, Japan

## Abstract

This study investigated the impact of paravalvular leakage (PVL) in relation to the different valve openings of the transcatheter aortic valve implantation (TAVI) valve using the fluid structure interaction (FSI) approach. Limited studies were found on the subject of FSI with regards to TAVI-PVL condition, which involves both fluid and structural responses in coupling interaction. Hence, further FSI simulation with the two-way coupling method is implemented to investigate the effects of hemodynamics blood flow along the patient-specific aorta model subjected to the interrelationship between PVL and the different valve openings using the established FSI software ANSYS 16.1. A 3D patient-specific aorta model is constructed using MIMICS software. The TAVI valve identical to Edward SAPIEN XT 26 (Edwards Lifesciences, Irvine, California), at different Geometrical Orifice Areas (GOAs), is implanted into the patient's aortic annulus. The leaflet opening of the TAVI valve is drawn according to severity of GOA opening represented in terms of 100%, 80%, 60%, and 40% opening, respectively. The result proved that the smallest percentage of GOA opening produced the highest possibility of PVL, increased the recirculatory flow proximally to the inner wall of the ascending aorta, and produced lower backflow velocity streamlines through the side area of PVL region. Overall, 40% GOA produced 89.17% increment of maximum velocity magnitude, 19.97% of pressure drop, 65.70% of maximum WSS magnitude, and a decrement of 33.62% total displacement magnitude with respect to the 100% GOA.

## 1. Introduction

Transcatheter Aortic Valve Implantation (TAVI) is one of the latest treatments of heart valve disease in nonoperable patients with severe aortic stenosis (AS) [1, 2]. It is recognized as the minimally invasive heart valve replacement for patients with high surgical risk and multimorbidity. Indeed, the paravalvular leakage (PVL) is highlighted as the serious complication after undergoing TAVI and thus received tremendous attention by many researchers worldwide [3–5].

According to Luu et al. [6], the PVL is referred to as a small opening between the aortic annulus and prosthetic valve, where the blood flowed through the uncovered portion of the stent frame. This complication occurred due to underexpansion of the prosthetic valve, undersizing, interference with stent expansion due the impingement of calcium nodules, and malpositioning of the valve [6, 7]. Lerakis et al. [8] stated that the PVL remains a frequent issue after implantation, which can be graded as mild (7.8%–40.8%), moderate (5%–36.9%), and severe (0.5%–13.6%). Hence, this serious complication leads to implication issues related to PVL after TAVI. Besides, the TAVI leaflets are made of glutaraldehyde-treated bovine pericardium (GLBP) or porcine pericardium [9], which can also result in the possibility of calcification and leaflet thickening. This consequence is similar to that observed in bioprosthetic valves in which heavily calcified stiff cusps may develop after years, thus leading to severe AS disease [10–12].

Furthermore, the emergence of today's technology, specifically on coupling techniques of clinical imaging such as magnetic resonance imaging (MRI), computed tomography (CT), and ultrasound Doppler imaging with numerical simulation, helps researchers to understand detailed behavior of blood flow, especially on the impact of stenosis development in patients' health [13–26]. Martin and Sun [9] performed the finite element analysis (FEA) on TAVI and bioprosthetic valves, where the authors compared leaflet fatigue of both valves under identical loading conditions. From their study, they noticed that TAVI devices showed lower durability for about 7.8 years compared to the bioprosthetic valve. Bianchi et al. [27] investigated the effect of crimping mechanics between the polymeric valve (Polynova, Inc) and Edwards SAPIEN valve and also the effect of different positioning of TAVI deployment using FEA. The results showed that the Polynova valve shows better results in withstanding the crimping stent compared to the SAPIEN valve with 48.23% reduction of maximum stress and 78.75% reduction of maximum Von Mises stress. Moreover, the results also supported that the existence of calcification deposits between the aortic valve wall and TAVI suboptimal valve affected the anchoring stent which led to the presence of gaps and PVL.

In spite of that, Basri et al. [20] studied behavior of blood flow along the aorta due to AS disease using computational fluid dynamics (CFD). From the simulation, the authors clearly proved that the severity of AS has disturbed the natural flow of blood into the carotid branches and increased the magnitude of maximum velocity to 13.7% compared to the normal aortic valve opening. Consequently, this leads to unequal distribution of blood supply into the important organs of the body [28]. Another study by Mao et al. [29] has developed a computational model predicting the severity of PVL after TAVI. In this study, FEA and CFD simulation were conducted separately. A nonlinear FE method was used to simulate the deployment of self-expandable CoreValve into a patient-specific aortic root with human aortic tissue properties. Then, CFD simulation was performed to investigate the impact factor of TAVI orientation, TAVI skirt shape, and deployment height effect on PVL. The results concluded that the TAVI orientation produced huge impact of PVL development as large as 40%. Moreover, the consequence of having small stent thickness compared to the aortic annulus size can lead to PVL complication, which estimated to be10 ml/beat. Bianchi et al. [30] also employed the similar techniques of FEA and CFD simulations solely in order to investigate the influence of procedural parameters on postdeployment hemodynamics of PVL. The results showed a good agreement between echocardiography data and CFD simulation in terms of PVL jet locations and overall PVL degree. Furthermore, the authors also discovered that the positioning and balloon overexpansion achieved reduction in PVL volume as high as 47%.

Nowadays, the growing technology of simulation has led to the development of fluid structure interaction (FSI) simulation which produced a superior coupling technique between FEA and CFD methods. This FSI technique has improved the responses between fluid flow and structural behavior as well as it influenced mimicking of the realistic model of human organs. Recently, several FSI studies on TAVI received tremendous attention by researchers from all over the world. Based on the previous literature studies, most of the numerical simulations related to the PVL focused on the relationship between PVL and the aortic valve tissue using FEA and CFD, separately. Limited studies were found on the subject of FSI with regards to TAVI-PVL condition, which involve both fluid and structural responses in coupling interaction.

Mao et al. [31] developed a novel fully coupled FSI model using the smoothed-particle hydrodynamics (SPH) method, whereby the authors carried out analysis in terms of structural failure of the stent and aortic wall. The study compared simulation between FE-only model vs FSI model in TAVI simulation. The results showed that the FSI model produced realistic leaflet dynamics deformation compared with the FE model. This is due to the accurate spatial and temporal loading conditions imposed on the leaflets. Obviously, this research mainly focused on the stress and strain distribution effects on the TAVI leaflet between FE and FSI simulations. It can be concluded that the FSI model produced 13–18% higher peak stresses than the FE model due to the effect of water hammering on the FSI model during the closing phase. On a different perspective, Basri et al. [5] performed FSI simulation of the TAVI valve to investigate the occurrence of PVL in regards to the blood flow's effect along the aorta. The authors proved that PVL resulted in a larger recirculatory flow above the valve, which may induce the formation of blood thrombosis in patients [32]. Thus, differential pressure inside the aorta may worsen the migration of the valve, causing another serious complication in regards to TAVI. The authors concluded that the undersized TAVI produced 21.18% of PVL, hence causing unequal distribution of mass flow rate, particularly at the aortic branch region.

In this work, another FSI simulation technique is implemented to investigate the interrelationship between PVL and the severity of leaflet calcification in terms of GOA opening with regards to the hemodynamics flow along the patient-specific aorta model. A 3D patient-specific aorta is created using MIMICS software, TAVI valve is drawn using CATIA, and FSI simulation is accomplished using ANSYS 16.1. The leaflet opening of TAVI is drawn according to GOA of 5.31 cm^2^, 4.25 cm^2^, 3.19 cm^2^, and 2.12 cm^2^ representing 100%, 80%, 60%, and 40% of the leaflet opening area, respectively. The simulation provides advantages for the medical expertise to foretell the blood flow's behavior in terms of blood velocity, pressure, and aorta displacement. The outcome of this research showed those impacts on the patient's health.

## 2. Methodology

### 2.1. Patient-Specific Aorta Model

The geometry of an aorta is obtained from gated clinical cardiac 64-slice CT scans provided by the National Heart Institute (IJN), Malaysia. The local ethics' committee has approved, and the patient has given informed consent for this study. A 71-year-old male diagnosed with severe AS is selected. The patient's annulus diameter is determined from the image data with the value of 27.3 mm. The DICOM format of the image is imported to MIMICS software (Materialise, Leuven, Belgium), and the 3D model is developed using the segmentation method. A detailed description of the segmentation process has been described in the previous study by Basri et al. [5].

### 2.2. Segmentation and 3D Model Development

**Figure 1:**
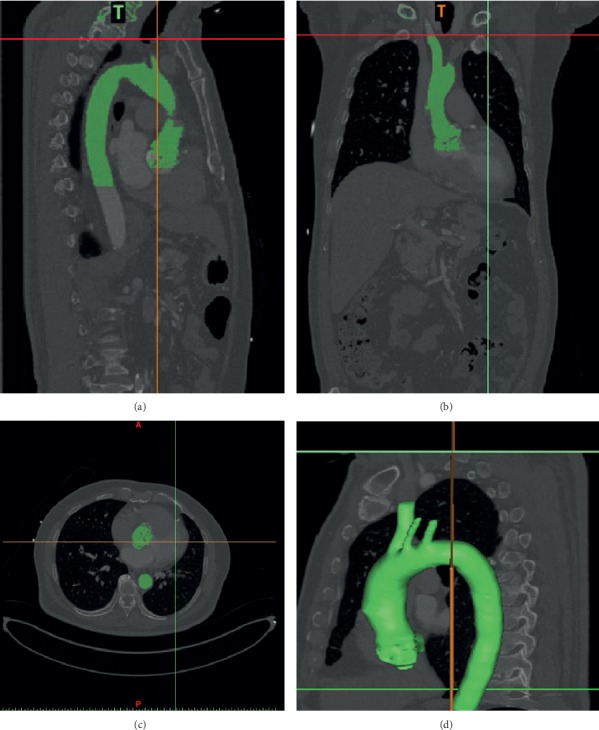
Aorta CT scan image from (a) sagittal (b) coronal (c) and axial planes. (d) 3D aorta model.

**Figure 2:**
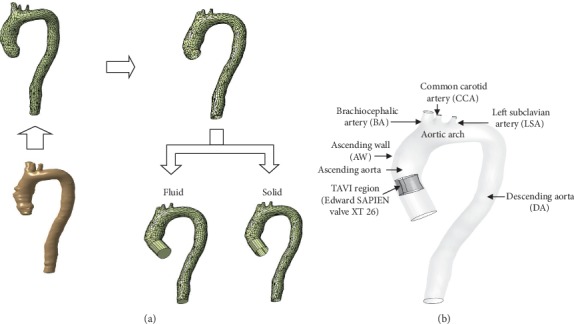
Process in developing the(a) 3D aorta model using CATIA and (b) 3D aorta model with valve location.

A 3D aorta model is developed based on pre-CT scan image data of the patient as shown in Figures 1(a)–1(d). However, the CT scan image data for post-TAVI implantation are not available. The obtained image is in the DICOM format (^∗^.dcm) and is imported later into the MIMICS software (Materialise Sdn. Bhd.) in order to produce a 3D image of the aorta model. In MIMICS, the obtained 2D image is developed and an accurate 3D anatomical model is constructed using the segmentation method. The complete 3D model of the aorta that consists of the ascending aorta, aortic arch, brachiocephalic artery, common carotid artery, left subclavian artery, and descending aorta is developed as shown in Figure 1(d). Then, the final 3D model of aorta geometry is converted into Stereo-Lithography format (^∗^.stl) before being exported to CATIA V5 software for the generation of a solid model (Figure 2(a)). Meanwhile, Figure 2(b) shows the 3D aorta model with valve location at the ascending aorta region.

### 2.3. TAVI Leaflet Opening

**Figure 3:**
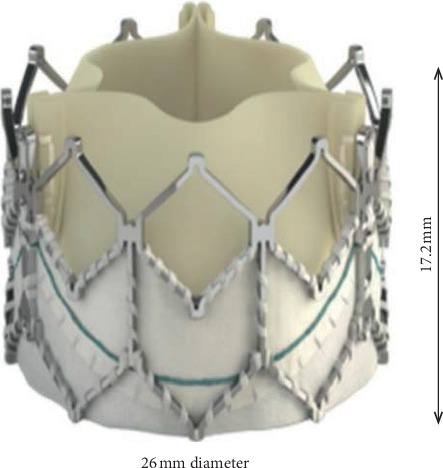
26 mm diameter of Sapien XT (Edward SAPIEN Aortic Valve; Edwards Lifesciences, Irvine, California).

The location of aortic valve placement is identified, and an annulus diameter of 27.3 mm is chosen as reference to redraw the TAVI valve. The 3D valve of 26 mm diameter is developed using the design data from the manufacturer, the Sapien XT (Edward SAPIEN Aortic Valve; Edwards Lifesciences, Irvine, California), as depicted in Figure 3 [33]. The undersized of 26 mm TAVI valve is selected for this study which represent the presence of PVL occured at the aortic annulus. In this study, the TAVI leaflet is drawn in four different valve openings representing the GOA in terms of percentage, which are 100%, 80%, 60%, and 40% as in Figure 3.

**Figure 4:**
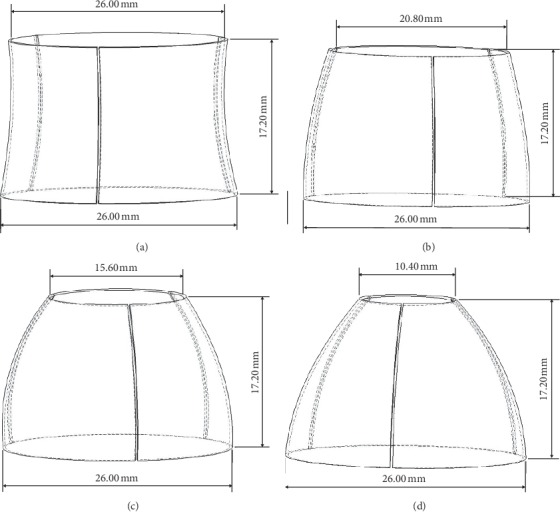
3D geometrical drawing of TAVI 26 Sapien XT with different GOA openings: (a) 100% GOA, (b) 80% GOA, (c) 60% GOA, and (d) 40% GOA.

**Figure 5:**
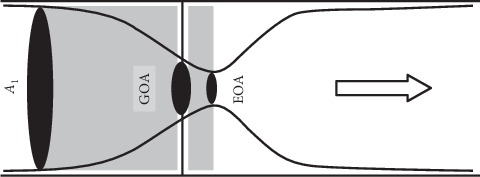
Relationship between GOA and EOA.

From Figure 4, it is observed that the lowest GOA percentage (40%) showed the mild valve stenosis, where higher net blood pressure loss through the valve occurs under this condition. In fact, this severity of stenosis can be referred to the calculation of aortic valve area (AVA) [34]. The AVA is estimated based on the valve inflow shape and cross-sectional area of the ascending aorta, which is represented as effective orifice area (EOA), as shown in Figure 5. According to Garcia et al. [34], the American College of Cardiology/American Heart Association (ACC/AHA) recommended the EOA/AVA value based on the classification of severity as follows: mild (>1.5 cm^2^); moderate (1.0 cm^2^–1.5 cm^2^); and severe (<1.0 cm^2^). Plus, GOA and EOA/AVA are directly proportional to the valve opening at low flow rates and tissue extensibility [11, 35, 36].

According to Garcia et al. [37], the relationship between GOA and EOA/AVA for the condition of flat, sharp-edge, and rigid stenosis, is shown as follows:

(1) $$\begin{array}{c} \frac{\text{EOA}}{\text{AVA}}=\text{GOA }{\left(1+\frac{\sqrt{2}}{2}\sqrt{1-\frac{GOA}{A_{1}}}\right)}^{-1}, \end{array}$$

where *A*_1_ is the inlet cross-sectional area of the aortic valve with the unit of mm^2^.

The severity of stenosis can be determined from the assessment of EOA/AVA, which basically referred to severity of AS. The EOA/AVA was assessed at the valve inflow shape and cross-sectional area of the ascending aorta. The relationship between GOA and EOA/AVA was directly proportional by means of valve opening at low flow rates and tissue extensibility. The severity of stenosis based on EOA/AVA was graded in Table 1 by using equation (1). With reference to Table 1, the smaller changes in valve openings or the reduction of GOA percentage may lead to calcification due to the decrement of GOA area. Hence, the 40% GOA is graded as mild AS, as referred to the EOA/AVA value given by ACC/AHA, which is 1.91 cm^2^. Therefore, by using the valve opening of GOA percentage representing the calcification, the assessment of PVL effects through FSI simulation can be carried out by comparing these valve openings in terms of relevant parameters such as velocity, pressure, WSS, and displacement.

### 2.4. Mesh-Dependence Study

**Figure 6:**
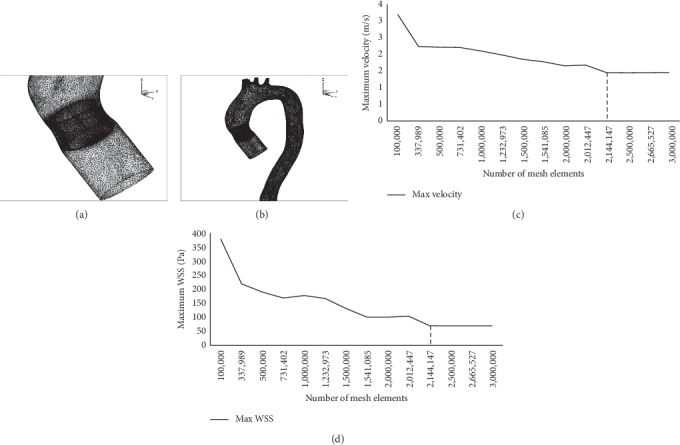
Result of meshes: (a) fluid domains and (b) solid domains. (c) Maximum velocity for fluid mesh dependence; (d) maximum total deformation for solid mesh dependence.

The grid dependency study was carried out for both fluid and solid domains, by comparing the mesh elements with the results of maximum velocity and maximum wall shear stress. The best selection of mesh for the fluid domain can be concluded to be approximately 2 million tetrahedral elements after being tested from 500,000 to 2.5 million number of mesh elements (Figures 6(a) and 6(c)). However, a mesh of approximately 75,000 quadrilateral elements is considered sufficient for the case of solid domain (Figures 6(b) and 6(d)). The dependency graph and mesh images are depicted in Figure 6.

### 2.5. Boundary Condition

#### 2.5.1. Fluid Dynamics Governing Equations

The keystone of fluid dynamics is the fundamental governing mathematical statements of the conservation laws of physics, which is the Navier–Stoke equation of fluid dynamics. This Navier–Stoke equation is essentially applied as the governing equation for the blood flow simulation taking into account the assumptions of turbulent, incompressible, homogenous, and Newtonian flow, which will be explained later. In this research, the energy equation and body forces were neglected as this study did not regard any thermal information. Hence, considering the incompressible fluid assumption, the mass and momentum conservation equations are as follows [38].

The continuity equation is

(2) $$\begin{array}{c} \nabla \cdot \overset{⟶}{V}\;=0. \end{array}$$

The momentum equation is

(3) α2Re∫∫∫v∂V⟶∂tdV⟶+∫∫∫vV⇀·∇V⇀dV⇀= −∫∫sp·dA+ 1Re∫∫sτ·dA,

where *τ* is the viscous stress tensor; *p* is the pressure; Re=2*RU*/*v* is the Reynolds number; *α*=*R*(*ω*/*v*)^1/2^ is the Womersley parameter; *U* is the maximum inlet velocity; *R* is the aorta inlet radius; *v* is the kinematic viscosity; and *ω* is the inlet pulse frequency (*ω*=2*πf*; *f* is the heart rate).

According to López et al. [38], the deviatoric stress tensor is related to the strain rate tensor which implies the proportionality tensor between stress and rate of strain. It defines the incompressible Newtonian fluid and satisfies the conditions of homogeneity. Thus, the algebraic equations that explain this relationship are written, as follows:

(4) $$\begin{array}{c} \tau =\mu \times \left(\dot{\gamma }\right)\dot{\gamma },\\ \dot{\gamma }=\left(\frac{\partial v_{i}}{\partial x_{j}}+\frac{\partial v_{j}}{\partial x_{i}}\right), \end{array}$$

where *μ* = viscosity and $\dot{\gamma }$ = strain rate.

Furthermore, in ANSYS Fluent, the conservation laws are applied to the finite volume method to solve the governing equations of the fluid, and hence, the discrete equations can be obtained. In this numerical solution, the computational domain is referred to as geometry of the region of interest, which is divided into some discrete points on each cell or smaller subregions known as control volumes. The conservation equations are evaluated and discretized, where each control volume equation is being solved through the integration of the governing equations using iterative methods. Consequently, the approximated values of each flow field variable such as pressures and velocities are obtained at each specific cell or point throughout the domain.

#### 2.5.2. Computational Fluid Dynamics Model

The CFD solved the governing Navier–Stokes equation of fluid motion. The governing equations of flow that were considered in this study are shown in equations (1) and (2).

**Figure 7:**
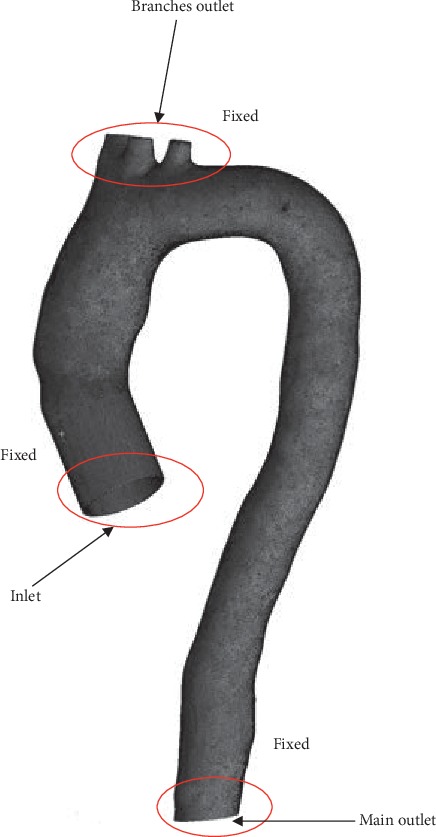
Boundary condition of fluid and solid domains.

**Figure 8:**
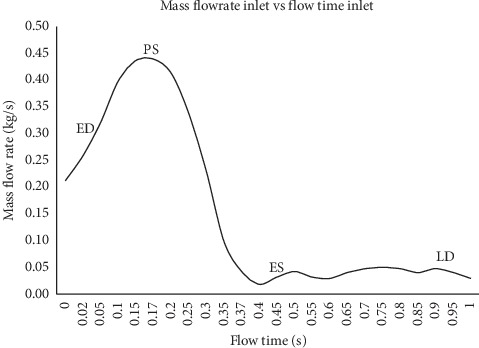
Mass flow rate inlet consists of early systole (ES), peak systole (PS), early diastole (ED), and late diastole (LD).

**Figure 9:**
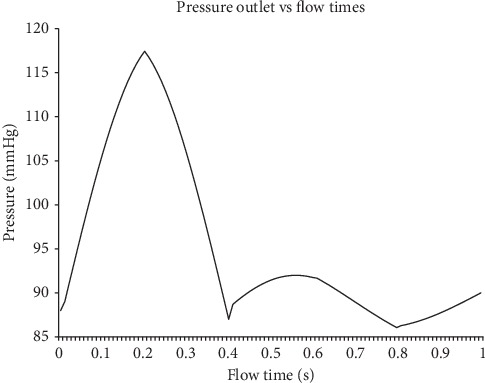
Blood pressure pulse that is used as output condition.

In this simulation, the mass flow rate and pressure are indicated as the respective inlet and outlet with the condition of pulsatile blood flow, according to Basri et al. [5] and Lantz et al. [39] (as in Figures 7–9). The inlet flow is assumed to be Newtonian and incompressible due to the higher relative shear rate ratio above 100 s^−1^ [40]. The values of blood density and viscosity are 1050 kg/m^3^ and 0.0035 Pa/s, respectively [41–43]. Using 27.3 mm of patient-specific annulus diameter and 0.732 m/s velocity at the peak systole (PS) state, the calculated Reynolds number is 5996.92. Therefore, the flow in this study is indicated as turbulent due to the obtained Reynolds number showed to be higher than 3000. For the turbulent model selection, k-*ω* shear stress transport (SST) is additionally used, according to Basri et al. [5], Lantz et al. [39], and Brown et al. [44].

The time-step dependency study is carried out at 0.1 s, 0.02 s, 0.01 s, 0.005 s, and 0.0025 s. The maximum velocity and maximum wall shear stress results are compared, and the time step of 0.01 s is finalized for the simulation, considering the small percentage difference of 6% between the time steps 0.01 s, 0.005 s, and 0.0025 s. The total simulation time is 3 s for three complete cardiac cycles, the solution converged at 10^−6^, and the final pulse is selected as the main mass flow rate inlet and pressure outlet [13, 45, 46]. It took about 168 hours to complete the simulation using the workstation with the configuration of Intel® Core™ i7-3520M CPU @ 2.90 GHz and 32 GB RAM. Hence, four time points of pulsatile flow are taken as the reference point in this study in order to observe the fluid flow behavior. Those points are early systole (ES), peak systole (PS), early diastole (ED), and late diastole (LD), as depicted in Figure 8.

#### 2.5.3. Finite Element Equations

Despite CFD equations for the fluid domains, the transient dynamic analysis in FEA is adopted in the structural dynamics to study the structural behavior under the application of loads, particularly for the solid domains [47]. The basic principle of this analysis is dividing the volume of a structure into a system of smaller elements called as finite elements. This conversion of finite elements is interconnected at the nodes, and the degrees of freedom are defined at these nodes. Then, the element force vector, mass matrix, and stiffness matrix with reference to the degrees of freedom are determined in a mesh by taking into account the relationship between the force-displacement and inertia force-acceleration for each element, as follows [47, 48]:

(5) $$\begin{array}{c} {\left(f_{s}\right)}_{e}=k_{e}u_{e},\\ {\left(f_{I}\right)}_{e}=m_{e}{\ddot{u}}_{e}, \end{array}$$

where **k**_*e*_ = element stiffness matrix, **m**_*e*_ = element mass matrix, **u**_*e*_ = displacement for the element, and ${\ddot{u}}_{e}$ = acceleration vector for the element.

Hence, the values of each element are assembled and connected to the global finite element in the form of transformation matrix (Boolean matrix contains zeroes and ones). These elements are allocated at the proper place of the global matrices and arranged based on the number of each element. The global stiffness, mass matrices, and applied force are evaluated as follows [47, 48]:

(6) $$\begin{array}{c} k=A_{e=1}^{N}k_{e},\\ m=A_{e=1}^{N}m_{e},\\ p\left(t\right)=A_{e=1}^{N}p_{e}\left(t\right), \end{array}$$

where *A* = operator responsible for assembly process, *N* = number of elements, and **p** **=** force vector in a function of time.

Thus, the basic governing equation of motion can be solved for **u**(*t*) based on the response of the system called nodal displacement values using iterative methods.

In this study, the material of the aortic wall is assumed to be linear elastic, which is adequate to determine the wall deformation. Hence, this research generates the FSI model by adopting the linear elastic model incompressible with isotropic Young's modulus, as studied by Lantz et al. [39]. On account of the assumptions of linearly elastic structure, internal damping is also considered. As shown in the computations by Kim et al. [49], the damping effect on the aortic wall is included to determine a more realistic dynamic simulation. Hence, the damping coefficient is added to ensure that the valve opens at a physiologically appropriate time scale as the given load is applied.

From the mentioned fundamental FE equation for solid domains, it is important to update the stiffness matrix for every time step. In this case, the transient dynamic involves the structural response of impulse load that acts on the structure with higher magnitude at a short interval of time. Using the Newmark method, the displacement is updated at every time interval followed by the stiffness matrix that is solved using a direct solver for every time step. Hence, the transient dynamic equation of the basic governing equation of motion for structure is written as follows [47]:

(7) $$\begin{array}{c} \left[M\right]\left\{\ddot{U}\right\}+\left[C\right]\left\{\dot{U}\right\}+\left[K\right]\left\{U\right\}=p\left(t\right), \end{array}$$

where *M* = structural mass matrix, $\ddot{U}$ = acceleration vector, *C* = structural damping matrix, $\dot{U}$ = velocity vector, *K* = structural stiffness matrix, *U* = displacement vector, and **p**(*t*) = force vector in a function of time.

#### 2.5.4. Finite Element Aorta Wall Model

In this study, the aortic wall is assumed to be linear elastic with the thickness of 1.5 mm (approximately 6% of the aortic diameter) [39], Poisson's ratio of 0.499, and density of 1080 kg/m^3^, which are the properties of the linear elastic material [43]. The isotropic Young's modulus of 1 MPa is chosen, as reported by [39, 41, 44, 48]. Besides that, the geometry is constrained in the axial direction at inlet, main outlet, and branch outlets as the fixed supports for aorta. A linear elastic support of 75 mmHg pressure was applied in the surrounding aorta wall to produce natural deflection of the aorta, whereby the wall was moved backward accordingly when the pressure decreased [5, 39]. Hence, it helped to eliminate the high-frequency modes of structural deformation [50]. The geometry is constrained in an axial direction at the inlet, branch outlet, and main outlet, as shown in Figure 7.

#### 2.5.5. Fluid Structure Interaction Coupling Equation

The interaction between fluid and solid in FSI simulation is coupled in two-way mode by the immersed boundary method in ANSYS 16.1 software [51]. FSI is always associated with complex problems. It involves the discretization of the mathematical model related to time and space as well as the time integration concerning both domains of fluid flow and structure in order to form the system of algebraic equations. The analysis consists of two-way load transfer at the interface, which is from the fluid and structure that leads to the change in boundary conditions. Moreover, the analysis is also required to update mesh at each step until the whole simulation is successful.

The conservation of mass equation in the fluid remains the same. However, the general momentum equation is unsuitable in transient analysis due to the frequent changes of solution domain every time, thus inducing the grid to be updated in order to change the flow boundary [52]. As mentioned previously, the ALE formulation is broadly used in the applications of vascular blood flow [53–55]. Based on the ALE grid formulation, a relative velocity that links the actual fluid velocity to the mesh velocity is taking the place of the actual fluid velocity with respect to a fixed mesh. Hence, this requires the grid to be updated all the time referring to the modified momentum equation, as shown in equation (8) for the denoted *i*th element:

(8) $$\begin{array}{c} \frac{\partial }{\partial t}\int_{\Omega }\rho \;\times \;\partial \Omega +\;\int_{S}\rho \;\times \;\left(v-v_{b}\right)\times n\times \partial S=\int_{S}\left(\tau_{ij\;}i_{j}-Pi_{i}\right)\;\times n\;\times \;\partial S+\;\int_{\Omega }b_{i}\times \partial \Omega , \end{array}$$

where *ρ* = density, *v* = velocity vector, *v*_*b*_ = grid velocity, *b* = body force given at time *t*, *τ* = stress tensor, *P* = pressure, ∂Ω = fluid domain, and ∂*S* = solid domain.

## 3. Result

### 3.1. Validation Study

The result of FSI normal aorta in terms of mass flow rate at ascending, velocity at ascending and descending aorta, and aorta deformation for different states is validated with the work done by Lantz et al. [39].

#### 3.1.1. Mass Flow Rate at Ascending Aorta

**Figure 10:**
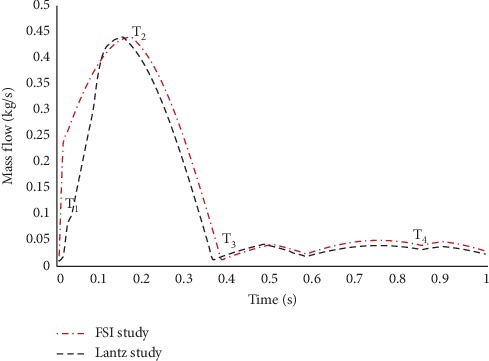
Mass flow rate at the ascending aorta.

The result of mass flow rate was acquired and validated by comparing it with the similar study by Lantz et al. [39], as shown in Figure 10.

From Figure 10, it can be observed that the graph of mass flow rate at the ascending aorta showed an almost similar pattern for both FSI study and the study by Lantz et al. [39] for the entire cardiac cycle. The graph of mass flow rate is started at the lowest point, where Lantz et al. [39] obtained an estimation of 0.020 kg/s and this FSI study obtained an estimation of 0.010 kg/s. The ES state of this FSI study is taken at the flow time of 0.02 s (T1), which showed the mass flow rate of 0.12 kg/s. Both studies reached the peak of PS state (T2) of 0.440 kg/s but at different flow time periods. Then, the graph showed a sudden drop towards ED state (T3) of 0.37 s with the estimation of mass flow rate of 0.042 kg/s. At the flow time of 0.9 s for LS state (T4), the mass flow rate obtained is estimated to be 0.048 kg/s slightly higher than that obtained by Lantz et al. [39]. Overall, the graph depicted the similar pattern of mass flow rate at the ascending aorta, although at different flow time periods. This is due to the effect of different patient-specific geometries between both data and also FSI effects. Yet, it is considered acceptable to be used for the FSI study.

#### 3.1.2. Velocity at the Ascending and Descending Aorta

**Figure 11:**
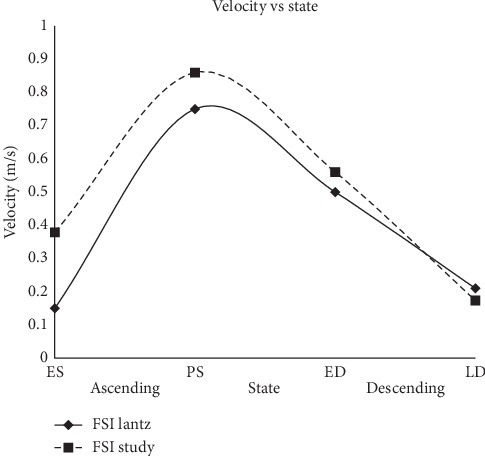
Velocity magnitude at the ascending and descending aorta.

The velocity at the ascending and descending aorta regions for different states is obtained and compared with the study by Lantz et al. [39], as depicted in Figure 11.

The graph of velocity magnitude at the ascending and descending aorta in Figure 11 showed an almost similar pattern for both studies of FSI and Lantz et al. [39]. It is noticed that the FSI study showed a higher velocity magnitude at ES, PS, and ED states but lower magnitude at LD state. At ES state, the average velocity at the ascending region of FSI simulation is stated to be 0.38 m/s, which is 2.5 times higher than the result by Lantz et al. [39] with 0.15 m/s. However, at PS state, the velocity of FSI simulation is 0.86 m/s with 17.05% higher than velocity obtained by Lantz et al. [39] of 0.75 m/s. However, the velocity magnitude showed a decrement at ED state for both studies. It is noticed that the velocity magnitude of Lantz et al. [39] is 0.50 m/s, with 10.71% lower than FSI simulation of 0.56 m/s. On the other hand, 19.05% lower velocity magnitude of FSI simulation is observed at LD state compared to Lantz et al. [39]. The velocity magnitude at this state is stated to be 0.17 m/s and 0.21 m/s for the respective FSI simulation and simulation by Lantz et al. [39]. The results between the current study and study by Lantz et al. [39] showed some differences due to the effect of different patient-specific geometries, where the current geometry has larger annulus diameter compared to the reference geometry. However, the results were acceptable due to the similar pattern and small percentage difference between both data especially towards LD state.

#### 3.1.3. Aorta Deformation

**Figure 12:**
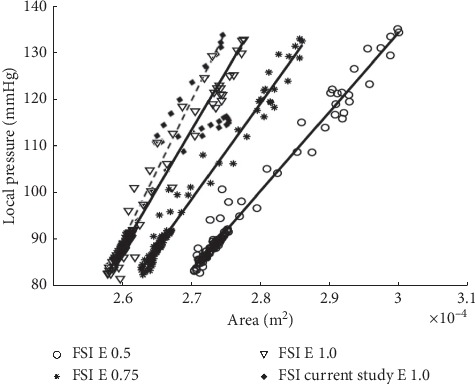
Graph of local pressure versus area.

As a fundamental, the aorta deformation is referred to Lantz et al. [39] by taking into account the relationship between local pressure and area of cross-sectional plane of the descending aorta region. The result of aorta deformation associated with local pressure with respect to the cross-sectional plane area by Lantz et al. [39] is as shown in Figure 12.

From Figure 12, it can be observed that the slope of the curve showed an almost similar pattern as the study by Lantz et al. [39]. However, the obtained graph showed a stiffer slope of the curve with higher Young's modulus. Moreover, the small difference range of the local pressure and area between both graphs is observed due to the different geometrical models of patient-specific data. Overall, the FSI study is considered acceptable and justifiable with the validation of existing research papers, particularly with regards to human physiology.

### 3.2. Velocity Flow Distribution of Different GOA Openings

**Figure 13:**
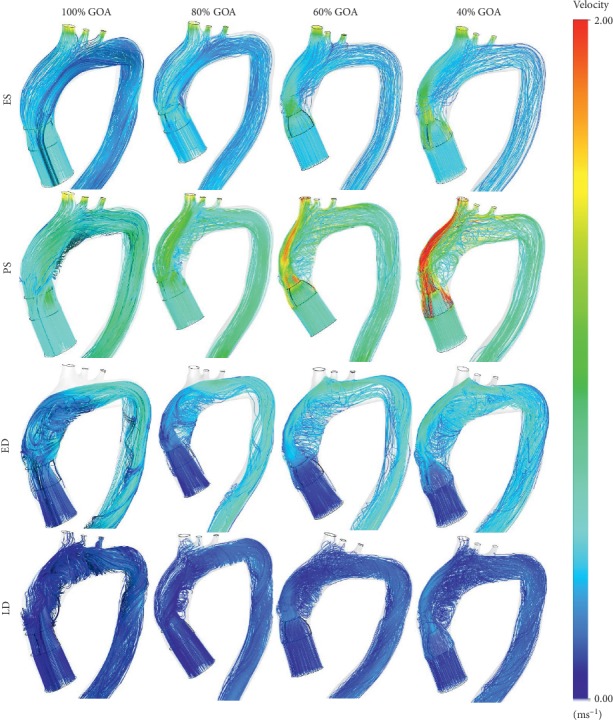
Anterior views of streamlines indicating the velocity magnitude.

**Figure 14:**
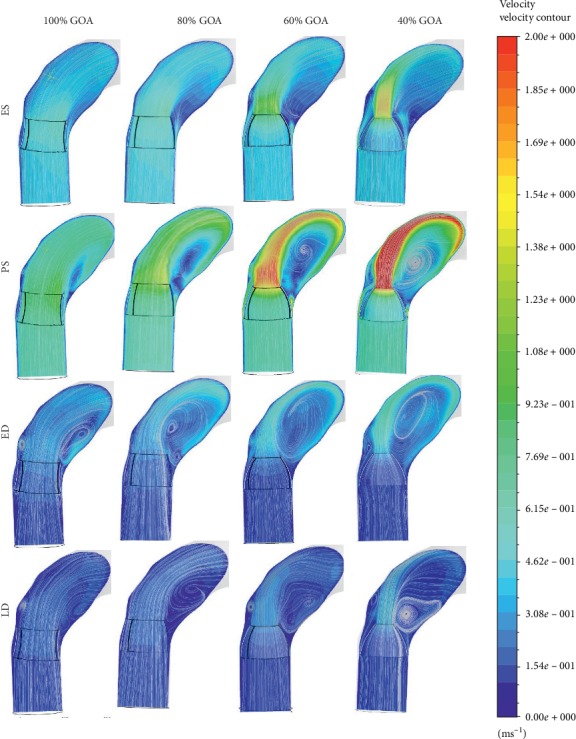
Velocity contour and streamlines cutting at *YZ* plane.

**Figure 15:**
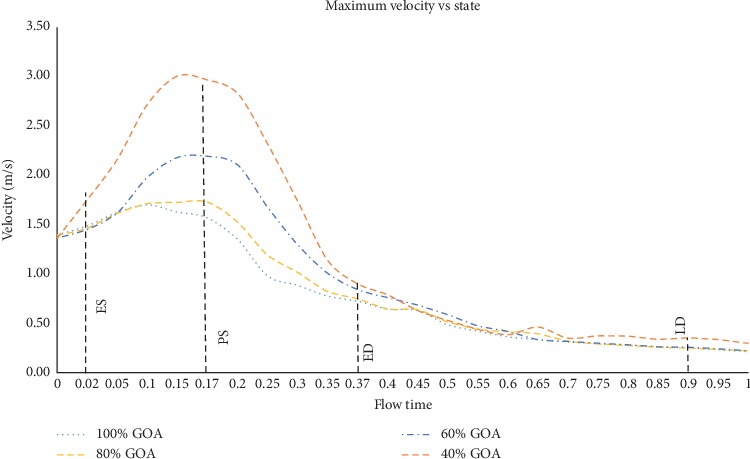
Maximum velocity of the 100% GOA, 80% GOA, 60% GOA, and 40% GOA for one cardiac cycle.

The velocity profile of 100% GOA, 80% GOA, 60% GOA, and 40% GOA is captured at four different states. The time instances of ES, PS, ED, and LD were 0.02 s, 0.17 s, 0.37 s, and 0.90 s for one cardiac cycle. The development and orientation of flow profiles for TAVI models are further discussed. The attribute of flow distribution can be explained in Figures 13–15 which represent velocity visualization for four opening conditions at different states.

In Figures 13 and 14, both velocity streamlines and contours showed some divergence of flow at the PVL region. In this situation, the velocity shows amplified streamlines at the centre of valve opening owing to the jet flow effect of the GOA openings. The most significant impact in terms of the flow distribution is 40% GOA during PS state compared to other states and conditions of opening. It is observed that the velocity streamline contour of 40% GOA performed the highest velocity especially at the PVL region and centre of valve opening, followed by 60% GOA, 80% GOA, and 100% GOA. Hence, the confluence of high velocity from the centre of valve opening and PVL region leads to the development of a huge helical flow at the inner wall of the ascending aorta and aortic arch regions. From the observation, 40% GOA showed the highest helical flow, followed by 60% GOA, 80% GOA, and 100% GOA. As shown in Figure 14, the highest velocity contour at the PVL region and highest recirculation flow were produced by 40% GOA, followed by 60% GOA, 80% GOA, and 100% GOA. This is due to the fact that smaller GOA opening performed higher velocity contour at the centre of valve opening and higher recirculation flow proximally to the inner wall of ascending aorta. Meanwhile, at ES state, it was noticed that 100% GOA and 80% GOA produced the backflow streamlines at ascending aorta and passed through the PVL region; however, it does not appear for 60% GOA and 40% GOA. The data in Figure 15 showed that the maximum velocity for the 40% GOA is stated to be 2.97 m/s, indicated as the highest compared to 60% GOA with 2.19 m/s, 80% GOA with 1.73 m/s, and 100% GOA with 1.57 m/s. On percentage difference, 40% GOA showed an increased 89.17% of maximum velocity compared to 100% GOA at the critical PS state.

### 3.3. Pressure Distribution of Different GOA Openings

**Figure 16:**
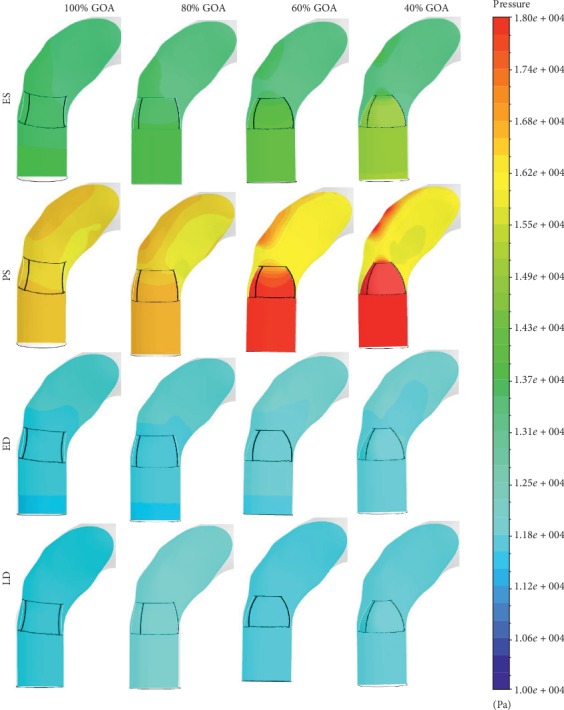
Pressure contour of 100% GOA, 80% GOA, 60% GOA, and 40% GOA at different states.

Meanwhile, the qualitative comparison of pressure contour can be obtained by referring to Figure 16. The pressure limit is set from 10 000 Pa to 18 000 Pa for a better comparison study. The red colour represents the highest pressure value, whereas the dark blue colour represents the lowest pressure value. According to Figure 16, the transition contour for 100% GOA, 80% GOA, and 60% GOA at ES state showed a small difference of green colour compared to 40% GOA. The transition contour of 40% GOA showed the major difference colour from light green to green, which is located in the downwards to upwards direction of the valve.

At PS state, 100% GOA is observed with dark yellow colour (indicated as higher pressure value) and light yellow (indicated as lower pressure value), where it is located downwards to the valve proximally to the outer wall and the inner wall of the ascending aorta, respectively. The transition of pressure contour became more conspicuous from 80% GOA to 40% GOA compared to 100% GOA. There was a high pressure value with red colour contour spotted downwards to the valve for 60% GOA and 40% GOA. Meanwhile, the pressure contour located upwards to the valve indicated the lowest pressure value of yellow colour for 40% GOA. This is due to the difficulties of the blood flow through the small GOA of the valve opening that produced high pressure downwards to the valve and low pressure upwards to the valve. This has led to a high pressure drop between the downwards and upwards direction of the valve opening. On the other hand, 40% GOA produced higher pressure at the outer wall of the ascending aorta compared to the others. Due to these circumstances, the blood distributions of 40% GOA are concentrated at the outer wall of the ascending aorta, hence leading to high pressure occurrence. On the other hand, 100% GOA and 80% GOA at ED state showed a noticeable pressure contour transition compared to 60% GOA and 40% GOA due to the large GOA area. At the final state of LD, it was noticed that there was no huge difference of pressure transition between these four conditions.

Hence, it can be noticed that the smaller GOA opening produced the higher pressure difference compared to the bigger GOA opening.  Table 2 indicates the percentage of pressure drop for each of the GOA openings. The 40% GOA showed the highest percentage of pressure drop with 19.97%, followed by 60% GOA, 80% GOA, and 100% GOA with, respectively, 10.00%, 3.62%, and 1.58%.

### 3.4. WSS Distribution of Different GOA Openings

**Figure 17:**
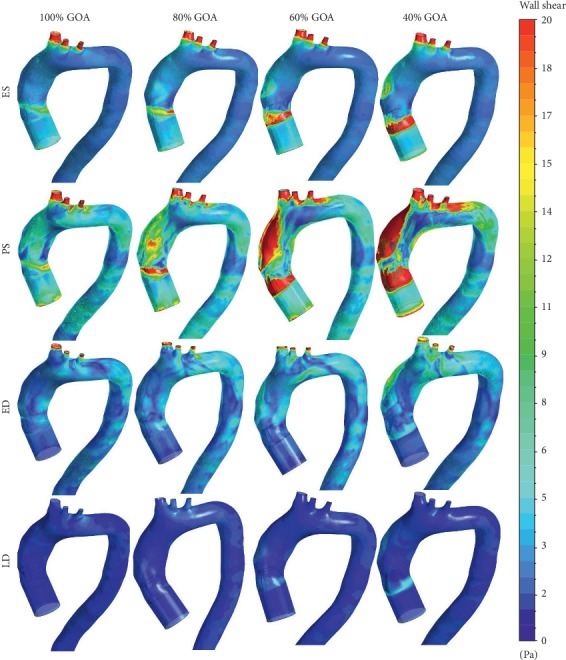
WSS contour at left view of 100% GOA, 80% GOA, 60% GOA, and 40% GOA at different states.

**Figure 18:**
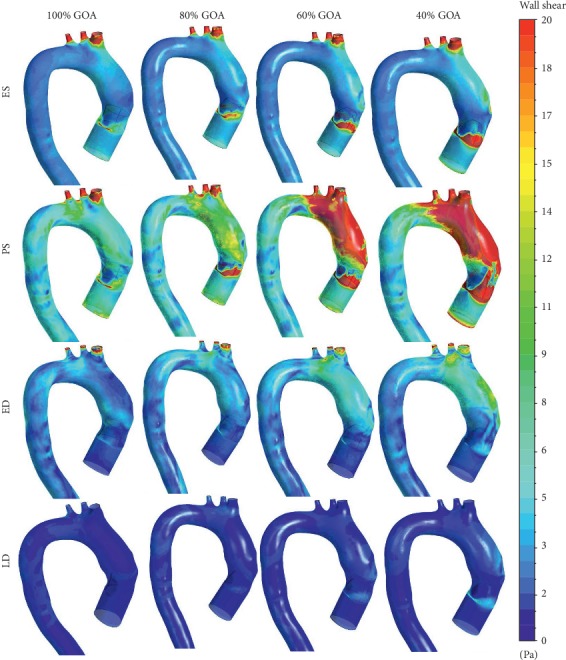
WSS contour at right view of 100% GOA, 80% GOA, 60% GOA, and 40% GOA at different states.

The WSS described in Figures 17 and 18 represent the visualization of WSS for all opening conditions at different states. From the simulation, the high value of WSS at the aortic wall may lead to the deformation of aorta, hence causing the rupture of aorta tissue. The WSS contour from the left and right views of 100% GOA, 80% GOA, 60% GOA, and 40% GOA at four different states is shown in Figures 17 and 18. The legend limit is standardized for all opening conditions and states with a maximum limit of 20 Pa.

**Figure 19:**
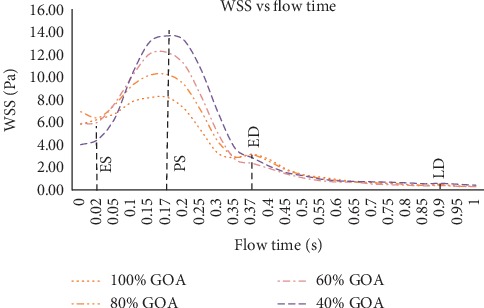
Mean WSS of 100% GOA, 80% GOA, 60% GOA, and 40% GOA.

Referring to Figures 17 and 18, it can be seen that the WSS contour of 100% GOA, 80% GOA, 60% GOA, and 40% GOA at ES state showed the huge differences at the ascending aorta proximally to the TAVI region (TR). The area of high WSS distributions at TR increased dramatically from 100% GOA to 40% GOA. For 100% GOA, it is noticed that only a small area of high WSS occurred at this region where the highest WSS value occurred proximally downwards to the valve. The area distributions of high WSS increased for 80% GOA to 40% GOA at the TR for both left and right views of the aorta due to the effects of high velocity of blood flow through the PVL region. Moreover, it is noticed that 40% GOA produced a higher WSS value at aortic wall (AW) region of the ascending aorta compared to the others. Quantitatively, the mean WSS in Figure 19 showed the results that 100% GOA produced the highest mean WSS with 6.25 Pa, followed by 80% GOA with 6.20 Pa, 60% GOA with 6.02 Pa, and 40% GOA with 4.47 Pa.

During the PS state, as shown in Figures 17 and 18, the WSS distributions increased tremendously compared to the previous conditions. It is noticed that the distributions of high WSS for 40% GOA and 60% GOA are higher than those of 80% GOA and 100% GOA. For 100% GOA, the high WSS occurred at the ascending aorta proximally downwards to the valve and also at the branches region with small size. Meanwhile, for 60% GOA and 40% GOA, the high WSS distributions mostly occurred at the surrounding of the TAVI valve region and also at the outer wall of ascending aorta. Hence, the high WSS distributions had occurred at the branches region with larger size than 80% GOA and 100% GOA. Quantitatively, the graph in Figure 19 exhibits that 40% GOA produced the highest mean WSS with 13.67 Pa, followed by 60% GOA with 12.22 Pa, 80% GOA with 10.26 Pa, and 100% GOA with 8.25 Pa.

During the ED, the graph in Figure 19 exhibits that 100% GOA produced the highest mean WSS with 3.16 Pa, followed by 80% GOA with 3.13 Pa, 40% GOA with 2.84 Pa, and 60% GOA with 2.35 Pa. Meanwhile, for LD state, 100% GOA produced the lowest mean WSS with 0.43 Pa, followed by 80% GOA with 0.49 Pa, 60% GOA with 0.54 Pa, and 40% GOA with 0.58 Pa. In terms of percentage difference, 40% GOA increased with 65.70% of maximum WSS value compared to 100% GOA at critical PS state.

### 3.5. Time Average Wall Shear Stress (TAWSS) Distribution

**Figure 20:**
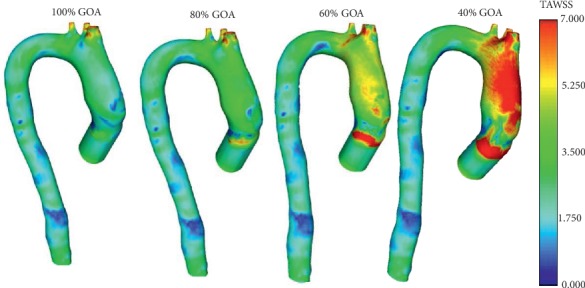
TAWSS of 100% GOA, 80% GOA, 60% GOA, and 40% GOA.

TAWSS represents the spatial variation of WSS [56, 57], as in Figure 20. The contour plot of the TAWSS distribution along the aorta is represented for 100% GOA, 80% GOA, 60% GOA, and 40% GOA. The highest TAWSS is found acting at the ascending aortic wall particularly for 40% GOA with 15.57 Pa, followed by 60% GOA, 80% GOA, and 100% GOA with 15.46 Pa, 11.17 Pa, and 7.73 Pa, respectively. This is due to the jet flow effect through the small opening of the TAVI valve and acting towards the ascending aortic wall, thus leading to high TAWSS magnitude especially for smaller GOA opening.

### 3.6. Total Mesh Displacement of Different GOA Openings

**Figure 21:**
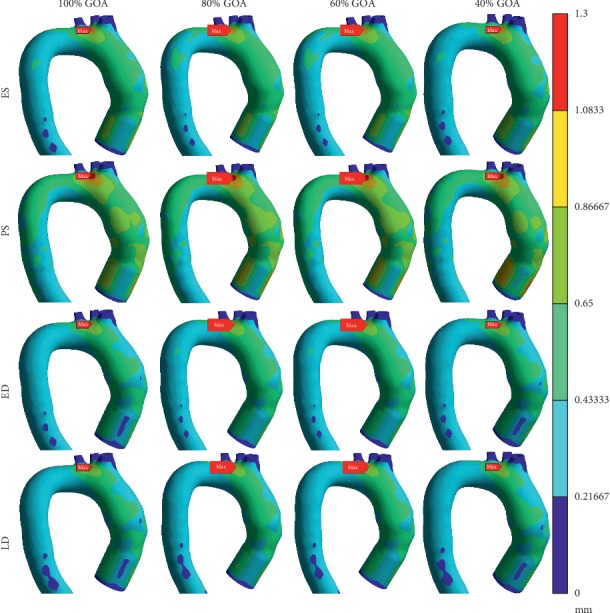
Total mesh displacement contour of 100% GOA, 80% GOA, 60% GOA, and 40% GOA at ES, PS, ED, and LD.

**Figure 22:**
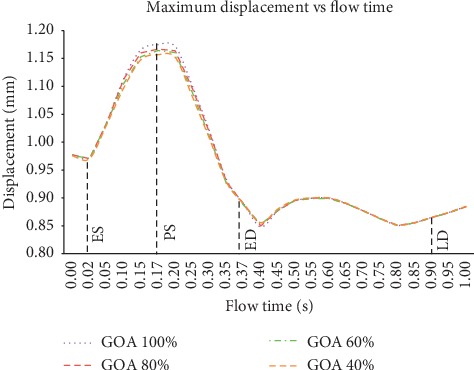
Maximum total mesh displacement of 100% GOA, 80% GOA, 60% GOA, and 40% GOA for a cardiac cycle.

The total mesh displacement contour and graph for all GOA opening are depicted in Figures 21 and 22, respectively. At ES state, the total mesh displacement of all GOA opening showed a small significant difference for the entire aorta but not at the ascending aorta. It is noticed that the high value of total mesh displacement at the ascending aorta downwards from the valve region increased from 100% GOA to 40% GOA with yellow colour contour. The results highlighted that four opening conditions shared the same location of maximum mesh displacement but different values. As shown in Figure 21, the highest maximum mesh displacement occurred at 100% GOA with 0.973 mm, followed by 80% GOA, 60% GOA, and 40% GOA with 0.970 mm each.

At PS state, the total mesh displacement distributions are amplified from the previous state. It is noticed that high value of the total mesh displacement occurred at the aortic arch region for all opening conditions. Moreover, high value of the total mesh displacement distributions at the ascending aorta downwards from the valve region increased from 100% GOA to 60% GOA and reached its peak at 40% GOA. In Figure 20, the maximum total mesh displacement occurred at aortic arch for 100% GOA, 80% GOA, and 40% GOA. Quantitatively, the highest maximum value of total mesh displacement occurred at 100% GOA with 1.175 mm followed by 80% GOA with 1.167 mm, 60% GOA with 1.164 mm, and 40% GOA with 1.157 mm, as shown in Figure 21.

At ED and LD states, there are no significant changes of high total mesh displacement between these four conditions as in Figure 20. 100% GOA, 80% GOA, 60% GOA, and 20% GOA shared the same locations of maximum total mesh displacement located at the aortic arch region. However, the quantitative data in Figure 21 proved that 40% GOA at ED state produced the highest value of total mesh displacement with 0.890 mm, followed by 100% GOA and 80% GOA with 0.889 mm each and 60% GOA with 0.887 mm. Meanwhile, the maximum mesh displacement of all GOA openings at LD state shared the same value of 0.865 mm. In terms of percentage difference, 40% GOA decreased with 33.62% of maximum total displacement along the aorta compared to 100% GOA at critical PS state.

## 4. Discussion

Throughout the results obtained from FSI simulation, the mechanical properties are compared to understand the fluid dynamics and structure deformation behavior on the patient-specific aorta model based on the GOA opening in relation to PVL complication. The flow behavior of velocity, pressure drop, WSS, and total displacement of each GOA opening are compared. The variance changes of these parameters proved the major impact from clinical complications, which can be explained as follows.

### 4.1. Development of Recirculation Flow Led to the Thrombus Formation

The reduction of GOA opening produced a huge recirculation flow through the PVL region, which is similar to the behavior of AS discussed in [58]. This phenomenon is proven when the confluence of high velocity from the centre of GOA opening and PVL region occurred due to the gradient change of blood velocity. Hence, the smallest GOA opening produced the huge recirculation flow. These circumstances may cause the development of thrombus at the aorta region, which led to the blood thrombosis, as supported by Stein et al. [59].

### 4.2. Effects of Pressure Drop on the Aorta Wall Collapse and Energy Losses

The smaller percentage of GOA opening with PVL has the higher possibility of significant pressure drop by means of the obstruction of blood flow. This consequence of pressure drop led to high pressure loss which augmented the flow resistance. Hence, this may cause the aorta wall to be collapsed [60–62]. From this study, the 40% GOA showed the highest percentage of pressure loss with 24.95% compared to the others, which burdened the left ventrical outflow tract (LVOT) significantly and led to the failure of heart [60, 63]. On the other hand, the high percentage of pressure drop enhanced the energy losses. This energy is dissipated due to the opening of thickened leaflets, as mentioned by Bluestein et al. [64]. As a matter of fact, lesser energy is produced at the aorta, which reduced the displacement of the aorta.

### 4.3. Development of Severe Leaflet Calcification and Aortic Rupture

In this study, the blood pressure from the LVOT is focused inside the area of TAVI valve before it occurs through the small area of the GOA opening. These circumstances caused a high pressure focused at the leaflet and also increased the WSS effects at the leaflet tip. Thus, this proves the consequence of severe leaflet calcification. Moreover, it is also observed that the smallest opening produced the highest pressure at the ascending wall of aorta due to the jet flow effects. If these circumstances continuously occurred, the aortic rupture has higher potential to appear at the ascending aortic wall due to the high WSS effects acting on the aorta tissue [19, 65, 66].

### 4.4. High WSS and TAWSS Lead to Platelet Activation and Damage of Endothelial Cells

The smaller percentage of GOA opening produced a higher value of WSS and TAWSS. These circumstances lead to platelet activation and form microparticles in native blood due to very high shear stress effects [67, 68]. In this study, the magnitude of TAWSS increased in parallel with the reduction of GOA opening, which is higher than that reported for the normal arterial wall which is in the range sof 1–7 Pa [56]. Moreover, this also led to the induced damage of endothelium due to the endothelial cell sensitivity and elevated levels of WSS and TAWSS changes [69–71].

### 4.5. Aortic Wall Displacement Effect due to the Disturbance of Blood Flow Distribution

The severe AS caused the disturbance of blood flow through the centre of the valve opening and PVL region, which can be seen from velocity flow perspective. From the comparison study, 40% GOA showed the highest magnitude of velocity flow due to the jet flow effects. On the other hand, these circumstances also disturbed the norm of the blood distribution along the aorta and have affected the development of aortic wall displacement, where the smaller GOA opening produced lower aortic wall displacement than the higher GOA opening. Consequently, the displacement of aortic wall due to the forces that the heart exerts on the aorta, lead to two circumstances; one is the large aortic root displacement may good in healthy aorta and secondly, it would be disaster for the case of stiffer aortic tissue that can cause the presence of ascending thoracic aortic aneurysm (ATAA) disease [72].

## 5. Conclusion

The hemodynamic effects of the GOA opening in relation to PVL diseases have been explored in this study. With the same material of TAVI leaflet and bioprosthetic valve, the potential of AS disease can be replicated in numerical simulation of GOA opening. The findings of this study hypothesized relatively the mechanisms of GOA opening in the PVL at the aorta region:

- The specific alterations in the flow field influenced the multicomplications of PVL diseases
- The development of GOA opening involved the interaction between the mechanical behavior of the blood flow and the biological processes occurring at the aorta region. Hence, the deteriorated aortic wall displacement is subjected to an elevated pressure regime, similar to the development of AS, before the implantation of TAVI.
- With the aid of computational simulation of FSI, the consequence of the GOA opening in relation to PVL in terms of severe implications toward the TAVI patient is comprehensively explained in this study.
- Based on the FSI simulation results, it can be concluded that 40% GOA increased with 89.17% of maximum velocity magnitude, 19.97% of pressure drop, and 65.70% of maximum WSS magnitude but decreased in total displacement magnitude with 33.62% with respect to the 100% GOA, respectively.
- Hence, the development of AS in PVL leads to the development of recirculation flow, thrombus formation, aorta wall collapse, energy losses, and development of severe leaflet calcification and aortic rupture.

## Figures and Tables

**Table 1:** GOA and EOA/AVA area for TAVI 26 mm Sapient XT.

GOA	100%	80%	60%	40%
GOA area	5.31 cm^2^	4.25 cm^2^	3.19 cm^2^	2.12 cm^2^
EOA/AVA	4.78 cm^2^	3.82 cm^2^	2.89 cm^2^	1.91 cm^2^

**Table 2:** Percentage of pressure drop for the 100% GOA, 80% GOA, 60% GOA, and 40% GOA at PS state.

GOA (%)	Pressure downward from valve (Pa)	Pressure upward from valve (Pa)	Pressure drop (Pa)	Pressure drop (%)
100	16469	16209	259	1.58
80	16730	16125	605	3.62
60	17859	16074	1785	10.00
40	19985	15994	3991	19.97

## Data Availability

The data used to support the findings of this study are included within the article and appendix. Raw data files of the models and simulations can be released upon application to the corresponding author (adiazriff@upm.edu.my).

## Nomenclature

TAVI: Transcatheter aortic valve implantation
PVL: Paravalvular leakage
FSI: Fluid structure interaction
ESV: Edward SAPIEN valve
AS: Aortic stenosis
SPH: Smoothed particle hydrodynamics
AVA: Aortic valve area
GOA: Geometrical orifice area
ATAA: Ascending thoracic aortic aneurysm
GLBP: Glutaraldehyde-treated bovine pericardium
MRI: Magnetic resonance imaging
CT: Computed tomography
FEA: Finite element analysis
CFD: Computational fluid dynamics
IJN: National Heart Institute
EOA: Effective orifice area
ACC/AHA: American College of Cardiology/American Heart Association.

## References

[1] Masson J.-B., Kovac J., Schuler G. (2009). Transcatheter aortic valve implantation. *JACC: Cardiovascular Interventions*.

[2] Fanning J. P., Platts D. G., Walters D. L., Fraser J. F. (2013). Transcatheter aortic valve implantation (TAVI): valve design and evolution. *International Journal of Cardiology*.

[3] Unbehaun A., Pasic M., Dreysse S. (2012). Transapical aortic valve implantation. *Journal of the American College of Cardiology*.

[4] Vasa-Nicotera M., Sinning J.-M., Chin D. (2012). Impact of paravalvular leakage on outcome in patients after transcatheter aortic valve implantation. *JACC: Cardiovascular Interventions*.

[5] Basri A. A., Zuber M., Zakaria M. S. (2016). The hemodynamic effects of paravalvular leakage using fluid structure interaction; transcatheter aortic valve implantation patient. *Journal of Medical Imaging and Health Informatics*.

[6] Luu J., Ali O., Feldman T. E., Price M. J. (2013). Percutaneous closure of paravalvular leak after transcatheter aortic valve replacement. *JACC: Cardiovascular Interventions*.

[7] Kodali S. K., Williams M. R., Smith C. R. (2012). Two-year outcomes after transcatheter or surgical aortic-valve replacement. *New England Journal of Medicine*.

[8] Lerakis S., Hayek S. S., Douglas P. S. (2013). Paravalvular aortic leak after transcatheter aortic valve replacement. *Circulation*.

[9] Martin C., Sun W. (2015). Comparison of transcatheter aortic valve and surgical bioprosthetic valve durability: a fatigue simulation study. *Journal of Biomechanics*.

[10] Baumgartner H. (2006). Hemodynamic assessment of aortic stenosis. *Journal of the American College of Cardiology*.

[11] Kurtz C. E., Otto C. M. (2010). Aortic stenosis. *Medicine*.

[12] Singhal P., Luk A., Butany J. (2013). Bioprosthetic heart valves: impact of implantation on biomaterials. *ISRN Biomaterials*.

[13] Jahangiri M., Saghafian M., Sadeghi M. R. (2015). Numerical study of turbulent pulsatile blood flow through stenosed artery using fluid-solid interaction. *Computational and Mathematical Methods in Medicine*.

[14] Khader S. M. A., Ayachit A., Raghuvir Pai B., Ahmed K. A., Rao V. R. K., Kamath S. G. FSI simulation of increased severity in patient specific common carotid artery stenosis.

[15] Zakaria M. S., Ismail F., Tamagawa M. (2019). A Cartesian non-boundary fitted grid method on complex geometries and its application to the blood flow in the aorta using OpenFOAM. *Mathematics and Computers in Simulation*.

[16] Azriff A., Johny C., Khader S. M. A., Raghuvir Pai B., Zuber M., Ahmed K. A. (2018). Numerical study of haemodynamics in abdominal aorta with renal branches using fluid–structure interaction under rest and exercise conditions. *International Journal of Recent Technology and Engineering (IJRTE)*.

[17] Sigüenza J., Pott D., Mendez S. (2018). Fluid-structure interaction of a pulsatile flow with an aortic valve model: a combined experimental and numerical study. *International Journal for Numerical Methods in Biomedical Engineering*.

[18] Febina J., Sikkandar M. Y., Sudharsan N. M. (2018). Wall shear stress estimation of thoracic aortic aneurysm using computational fluid dynamics. *Computational and Mathematical Methods in Medicine*.

[19] Dabagh M., Vasava P., Jalali P. (2015). Effects of severity and location of stenosis on the hemodynamics in human aorta and its branches. *Medical & Biological Engineering & Computing*.

[20] Basri A. A., Zuber M., Zakaria M. S. (2016). The effects of aortic stenosis on the hemodynamic flow properties using computational fluid dynamics. *International Journal of Heat and Fluid Flow*.

[21] Basri A. A., Khader A. S. M., Johny C. J. (2018). Numerical study of haemodynamics behaviour in normal and single stenosed renal artery using fluid-structure interaction. *Journal of Advanced Research in Fluid Mechanics and Thermal*.

[22] Gsell M. A. F., Augustin C. M., Prassl A. J. (2018). Assessment of wall stresses and mechanical heart power in the left ventricle: finite element modeling versus laplace analysis. *International Journal for Numerical Methods in Biomedical Engineering*.

[23] Zakaria M. S., Ismail F., Tamagawa M. (2016). Numerical analysis using a fixed grid method for cardiovascular flow application. *Journal of Medical Imaging and Health Informatics*.

[24] Zakaria M. S., Ismail F., Tamagawa M. (2017). Review of numerical methods for simulation of mechanical heart valves and the potential for blood clotting. *Medical & Biological Engineering & Computing*.

[25] Zakaria M. S., Ismail F., Tamagawa M. (2018). Computational fluid dynamics study of blood flow in aorta using OpenFOAM. *Journal of Advanced Research in Fluid Mechanics and Thermal*.

[26] Khader S. M. A., Azriff A., Pai R. (2018). Haemodynamics study in subject-specific abdominal aorta with renal bifurcation using CFD-a case study. *Journal of Advanced Research in Fluid Mechanics and Thermal*.

[27] Bianchi M., Ghosh R. P., Marom G., Slepian M. J., Bluestein D. Simulation of Transcatheter Aortic Valve Replacement in patient-specific aortic roots: effect of crimping and positioning on device performance.

[28] Basri A. A., Zubair M., Aziz A. F. A., Ali R. M., Tamagawa M., Ahmad K. A. Computational fluid dynamics study of the aortic valve opening on hemodynamics characteristics.

[29] Mao W., Wang Q., Kodali S., Sun W. (2018). Numerical parametric study of paravalvular leak following a transcatheter aortic valve deployment into a patient-specific aortic root. *Journal of Biomechanical Engineering*.

[30] Bianchi M., Marom G., Ghosh R. P. (2019). Patient-specific simulation of transcatheter aortic valve replacement: impact of deployment options on paravalvular leakage. *Biomechanics and Modeling in Mechanobiology*.

[31] Mao W., Li K., Sun W. (2016). Fluid-structure interaction study of transcatheter aortic valve dynamics using smoothed particle hydrodynamics. *Cardiovascular Engineering and Technology*.

[32] Celestin C., Guillot M., Ross-Ascuitto N., Ascuitto R. (2015). Computational fluid dynamics characterization of blood flow in central aorta to pulmonary artery connections: importance of shunt angulation as a determinant of shear stress-induced thrombosis. *Pediatric Cardiology*.

[33] Kasel A. M., Cassese S., Bleiziffer S. (2013). Standardized imaging for aortic annular sizing. *JACC: Cardiovascular Imaging*.

[34] Garcia D., Kadem L. (2006). What do you mean by aortic valve area: geometric orifice area, effective orifice area, or gorlin area?. *The Journal of Heart Valve Disease*.

[35] Burwash I. G., Thomas D. D., Sadahiro M. (1994). Dependence of Gorlin formula and continuity equation valve areas on transvalvular volume flow rate in valvular aortic stenosis. *Circulation*.

[36] Chambers J. B., Sprigings D. C., Cochrane T. (1992). Continuity equation and Gorlin formula compared with directly observed orifice area in native and prosthetic aortic valves. *Heart*.

[37] Garcia D., Pibarot P., Landry C. (2004). Estimation of aortic valve effective orifice area by Doppler echocardiography: effects of valve inflow shape and flow rate. *Journal of the American Society of Echocardiography*.

[38] López A. G., Reyes I. P., Villa A. L., Aguilar R. O. V. (2016). Stochastic simulation for couette flow of dilute polymer solutions using hookean dumbbells. *Recent Advances in Fluid Dynamics with Environmental Applications*.

[39] Lantz J., Renner J., Karlsson M. (2011). Wall shear stress in a subject specific human aorta - influence of fluid-structure interaction. *International Journal of Applied Mechanics*.

[40] Manimaran R. (2011). CFD simulation of non-Newtonian fluid flow in arterial stenoses with surface irregularities. *World Academy of Science, Engineering and Technology*.

[41] Gao F., Watanabe M., Matsuzawa T. (2006). Stress analysis in a layered aortic arch model under pulsatile blood flow. *BioMedical Engineering OnLine*.

[42] Marom G., Kim H. S., Rosenfeld M., Raanani E., Haj-Ali R. Effect of asymmetry on hemodynamics in fluid-structure interaction model of congenital bicuspid aortic valves.

[43] Marom G., Kim H.-S., Rosenfeld M., Raanani E., Haj-Ali R. (2013). Fully coupled fluid-structure interaction model of congenital bicuspid aortic valves: effect of asymmetry on hemodynamics. *Medical & Biological Engineering & Computing*.

[44] Brown S., Wang J., Ho H., Tullis S. (2013). Numeric simulation of fluid-structure interaction in the aortic arch. *Computational Biomechanics for Medicine*.

[45] Jahangiri M., Saghafian M., Sadeghi M. R. (2015). Effects of non-Newtonian behavior of blood on wall shear stress in an elastic vessel with simple and consecutive Stenosis. *Biomedical and Pharmacology Journal*.

[46] Jahangiri M., Saghafian M., Sadeghi M. R. (2017). Numerical simulation of non-Newtonian models effect on hemodynamic factors of pulsatile blood flow in elastic stenosed artery. *Journal of Mechanical Science and Technology*.

[47] Raja R. S. (2012). *Coupled Fluid Structure Interaction Analysis on a Cylinder Exposed to Ocean Wave Loading*.

[48] Chopra A. K. (2007). *Dynamics of Structures : Theory and Applications to Earthquake Engineering*.

[49] Kim H., Lu J., Sacks M. S., Chandran K. B. (2008). Dynamic simulation of bioprosthetic heart valves using a stress resultant shell model. *Annals of Biomedical Engineering*.

[50] Takizawa K., Moorman C., Wright S., Christopher J., Tezduyar T. E. (2010). Wall shear stress calculations in space-time finite element computation of arterial fluid-structure interactions. *Computational Mechanics*.

[51] Hou G., Wang J., Layton A. (2012). Numerical methods for fluid-structure interaction - a review. *Communications in Computational Physics*.

[52] Ferziger J. H., Peric M. (2002). *Computational Methods for Fluid Dynamics*.

[53] Degroote J., Bathe K.-J., Vierendeels J. (2009). Performance of a new partitioned procedure versus a monolithic procedure in fluid-structure interaction. *Computers & Structures*.

[54] Fernández M. Á., Moubachir M. (2005). A Newton method using exact jacobians for solving fluid-structure coupling. *Computers & Structures*.

[55] Formaggia L., Nobile F. (2004). Stability analysis of second order time accurate schemes for ALE-FEM. *Computer Methods in Applied Mechanics and Engineering*.

[56] Wan Ab Naim W. N., Ganesan P. B., Sun Z., Osman K., Lim E. (2014). The impact of the number of tears in patient-specific Stanford type b aortic dissecting aneurysm: CFD simulation. *Journal of Mechanics in Medicine and Biology*.

[57] Wan Ab Naim W. N., Ganesan P. B., Sun Z. (2018). Flow pattern analysis in type B aortic dissection patients after stent-grafting repair: comparison between complete and incomplete false lumen thrombosis. *International Journal for Numerical Methods in Biomedical Engineering*.

[58] Flachskampf F. A., Weyman A. E., Guerrero J. L., Thomas J. D. (1990). Influence of orifice geometry and flow rate on effective valve area: an in vitro study. *Journal of the American College of Cardiology*.

[59] Stein P. D., Sabbah H. N. (1976). Turbulent blood flow in the ascending aorta of humans with normal and diseased aortic valves. *Circulation Research*.

[60] Keshavarz-Motamed Z., Garcia J., Pibarot P., Larose E., Kadem L. (2011). Modeling the impact of concomitant aortic stenosis and coarctation of the aorta on left ventricular workload. *Journal of Biomechanics*.

[61] Ku D. N., Zeigler M. N., Downing J. M. (1990). One-dimensional steady inviscid flow through a stenotic collapsible tube. *Journal of Biomechanical Engineering*.

[62] Tang D., Yang C., Kobayashi S., Zheng J., Vito R. P. (2003). Effect of stenosis asymmetry on blood flow and artery compression: a three-dimensional fluid-structure interaction model. *Annals of Biomedical Engineering*.

[63] Brickner M. E., Hillis L. D., Lange R. A. (2000). Congenital heart disease in adults. *New England Journal of Medicine*.

[64] Bluestein D., Einav S., Einavt S., Transfer H. (1995). The effect of varying degrees of stenosis on the characteristics of turbulent pulsatile flow through heart valves. *Journal of Biomechanics*.

[65] Mori D., Yamaguchi T. (2002). Computational fluid dynamics modeling and analysis of the effect of 3-D distortion of the human aortic arch. *Computer Methods in Biomechanics and Biomedical Engineering*.

[66] Wolters B. J. B. M., Rutten M. C. M., Schurink G. W. H., Kose U., De Hart J., Van De Vosse F. N. (2005). A patient-specific computational model of fluid-structure interaction in abdominal aortic aneurysms. *Medical Engineering & Physics*.

[67] Sherif M. A., Abdel-Wahab M., Awad O. (2010). Early hemodynamic and neurohormonal response after transcatheter aortic valve implantation. *American Heart Journal*.

[68] Sakariassen K. S., Holme P. A., Ørvim U., Barstad R. M., Solum N. O., Brosstad F. R. (1998). Shear-induced platelet activation and platelet microparticle formation in native human blood. *Thrombosis Research*.

[69] Leuprecht A., Kozerke S., Boesiger P., Perktold K. (2003). Blood flow in the human ascending aorta: a combined MRI and CFD study. *Journal of Engineering Mathematics*.

[70] Nabaei M., Fatouraee N. (2012). Computational modeling of formation of a cerebral aneurysm under the influence of smooth muscle cell relaxation. *Journal of Mechanics in Medicine and Biology*.

[71] Sheard G. J. (2009). Flow dynamics and wall shear-stress variation in a fusiform aneurysm. *Journal of Engineering Mathematics*.

[72] Mendez V., Di Giuseppe M., Pasta S. (2018). Comparison of hemodynamic and structural indices of ascending thoracic aortic aneurysm as predicted by 2-way FSI, CFD rigid wall simulation and patient-specific displacement-based FEA. *Computers in Biology and Medicine*.

